# Role of acetylcholine spasm provocation test as a pathophysiological assessment in nonobstructive coronary artery disease

**DOI:** 10.1007/s12928-020-00720-z

**Published:** 2020-10-27

**Authors:** Satoru Suzuki, Koichi Kaikita, Eiichiro Yamamoto, Hideaki Jinnouchi, Kenichi Tsujita

**Affiliations:** 1 Department of Cardiovascular Medicine and Center for Metabolic Regulation of Healthy Aging, Graduate School of Medical Sciences, Kumamoto University, 1-1-1 Honjo, Chuo-ku, Kumamoto, 860-8556 Japan; 2 Diabetes Center, Jinnouchi Hospital, 6-2-3 Kuhonji, Chuo-ku, Kumamoto, 862-0976 Japan

**Keywords:** Coronary spasm, Acetylcholine provocation test, Nonobstructive coronary artery disease, Microvascular spasm

## Abstract

Coronary angiography (CAG) sometimes shows nonobstructive coronary arteries in patients with suspected angina or acute coronary syndrome (ACS). The high prevalence of nonobstructive coronary artery disease (CAD) in those patients has recently been reported not only in Japan but also in Western countries, and is clinically attracting attention. Coronary spasm is considered to be one of the leading causes of both suspected stable angina and ACS with nonobstructive coronary arteries. Coronary spasm could also be associated with left ventricular dysfunction leading to heart failure, which could be improved following the administration of calcium channel blockers. Because we rarely capture spontaneous attacks of coronary spasm with electrocardiograms or Holter recordings, an invasive diagnostic modality, acetylcholine (ACh) provocation test, can be useful in detecting coronary spasm during CAG. Furthermore, we can use the ACh-provocation test to identify high-risk patients with coronary spasm complicated with organic coronary stenosis, and then treat with intensive care. Nonobstructive CAD includes not only epicardial coronary spasm but also microvascular spasm or dysfunction that can be associated with recurrent anginal attacks and poor quality of life. ACh-provocation test could also be helpful for the assessment of microvascular spasm or dysfunction. We hope that cardiologists will increasingly perform ACh-provocation test to assess the pathophysiology of nonobstructive CAD.

## Introduction

**Fig.1:**
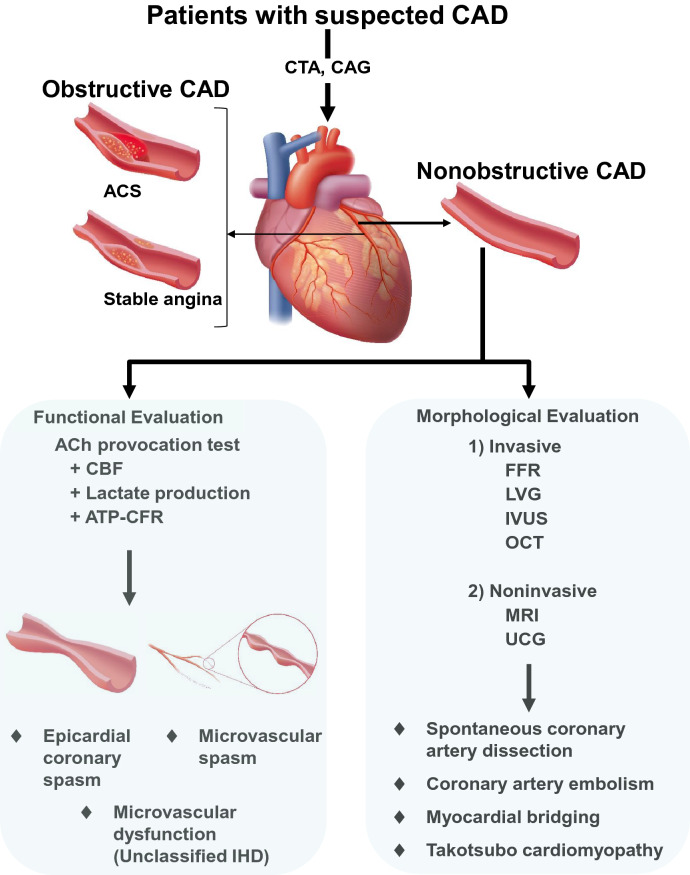
Diagnostic flowchart of functional evaluation using ACh-provocation test in nonobstructive CAD patients. *CAD* coronary artery disease, *CTA* computed tomography angiography, *CAG* coronary angiography, *ACS* acute coronary syndrome, *Ach* acetylcholine, *CBF* coronary blood flow, *ATP-CFR* adenosine triphosphate-induced coronary flow reserve, *IHD* ischemic heart disease, *FFR* fractional flow reserve, *LVG* left ventriculography, *IVUS* intravascular ultrasound, *OCT* optical coherence tomography, *MRI* magnetic resonance imaging; UCG, ultrasonic echocardiography

We often perform percutaneous coronary intervention (PCI) with coronary stent implantation for significant organic coronary artery stenosis with induced myocardial ischemia. PCI has become a useful treatment for relief of chest symptom in patients with significant coronary organic stenosis. However, coronary angiography (CAG) sometimes shows nonobstructive coronary arteries that do not need revascularization in patients with suspected angina [1–11]. Indeed, more than 50% of stable patients with suspected angina undergoing CAG had angiographically nonobstructive coronary arteries that did not require revascularization [4, 10]. In addition, despite the high prevalence of obstructive coronary artery disease (CAD) in acute myocardial infarction (AMI), up to 13% of AMI patients had nonobstructive CAD (myocardial infarction with nonobstructive coronary arteries [MINOCA]) [6, 7, 12–14]. This high prevalence of nonobstructive CAD has recently been reported not only in Japan but also in Western countries [6, 10, 15, 16]. Thus, nonobstructive CAD is attracting clinical attention because it may be associated with future cardiovascular events [1, 2, 4–7, 17]. Coronary spasm is considered to be one of the leading causes of both stable angina with nonobstructive CAD and MINOCA (the others being microvascular spasm, spontaneous coronary artery dissection, coronary artery embolism, myocardial bridging, and Takotsubo cardiomyopathy) [1, 5–7, 10, 11, 18, 19] (Fig. 1).

Yasue and coworkers previously reported that the intracoronary injection of acetylcholine (ACh) is a useful test for the provocation of coronary spasm [20–23]. Since these reports, angina pectoris caused by coronary spasm is now referred to as “coronary spastic angina” or “vasospastic angina” [24–35], and variant angina is regarded as one aspect of the wide spectrum of myocardial ischemic syndromes caused by coronary spasm [24].

Although the etiologic mechanisms of stable angina and acute coronary syndrome (ACS) in patients with nonobstructive CAD remain obscure, coronary spasm can play an important role in the pathogenesis of these diseases [1, 5–7, 16, 19, 24, 25, 36, 37]. Recent studies reported that coronary spasm could also be associated with left ventricular dysfunction leading to heart failure [38–41]. ACh-provocation test might be useful as a diagnostic tool of nonobstructive CAD, and might play a key role in the assessment of its pathophysiology [1, 5–7, 16, 21, 22, 24, 42].

In this review article, we discuss the significance of ACh-provocation test as a tool for pathophysiological evaluation in nonobstructive CAD including coronary spasm.

## Use of ACh in the coronary spasm provocation test

Coronary spasm usually occurs at rest, particularly from midnight to early morning [24–27]. Coronary spasm can be induced even by mild exercise in the early morning but is not usually induced in the afternoon even by strenuous exercise [24, 31–33]. Based on these reports, a circadian rhythm can be associated with the activity of coronary spasm, although the precise mechanisms of circadian variation in coronary spasm have not yet been elucidated. Our previous studies reported that coronary spasm can be induced by subcutaneous injection of methacholine, an analog of ACh that stimulates the parasympathetic nervous system, and that this attack can be suppressed by the parasympatholytic agent atropine, in patients with variant angina [22, 33, 43–47]. Activity of the parasympathetic nervous system is enhanced at rest and is suppressed by exercise [48]. At that time, we hypothesized that activity of the parasympathetic nervous system might be involved in the pathogenesis of variant angina or coronary spasm, and showed that the intracoronary injection of ACh could be useful for inducing coronary spasm [22, 23].

ACh usually causes vasodilation by releasing nitric oxide from the endothelium, and intracoronary injection of ACh in humans provokes coronary vasodilation in young healthy subjects, whereas it causes vasoconstriction in patients with coronary endothelial dysfunction such as coronary atherosclerosis [49–51]. The coronary arteries in patients with coronary spasm are highly sensitive to the vasoconstrictor effect of intracoronary injection of ACh, resulting in spasm [52]. Our previous reports showed that the sensitivity and specificity of the intracoronary injection of ACh were very high in variant angina and that multivessel coronary spasm was often observed in patients with variant angina following intracoronary injection of ACh [20, 21]. ACh-provoked coronary spasm is of short duration, probably because ACh is rapidly degraded by acetylcholinesterase in vivo [22]. In addition, previous studies reported low rates of complications with ACh-provocation test during CAG [53–55]. Thus, ACh-provocation test could be a safe and reliable method to diagnose coronary spasm during CAG [8, 24, 25, 47, 53–61].

Nonobstructive CAD includes not only angina with epicardial coronary spasm but also microvascular spasm [4–7, 62]. However, the coronary microcirculation is not easily visualized, while the epicardial coronary arteries are easily visualized by CAG or computed tomography angiography (CTA). Microvascular spasm or dysfunction can be assessed partially from the results of both coronary blood flow (CBF) during ACh-provocation test and adenosine triphosphate-induced coronary flow reserve (ATP-CFR), which is a non-endothelium-dependent coronary reactivity test [4, 10, 63–67]. The ACh-provocation test can be useful to assess the comprehensive pathophysiology of total coronary arteries including epicardial coronary arteries and microvascular lesions.

Ovisot^®^ (ACh chloride) was approved as an inducer of coronary spasm during CAG in 2017 in Japan. This approval will lead to more testing for provocation of coronary spasm in Japan, adaptation of the treatment based on the patient's progress, and detailed pathophysiological evaluation of nonobstructive CAD including coronary spasm.

In addition to ACh, ergonovine (ER) is also useful for pharmacological provocation of coronary spasm. According to the Japanese Circulation Society (JCS) Guidelines [25], the intracoronary injection of ER provocation test during CAG is also recommended in patients with suspected coronary spasm. In the coronary arteries of patients with coronary spasm, it is thought that intracoronary injection of ACh can provoke coronary spasm via muscarinic receptors, whereas intracoronary injection of ER can provoke it via serotonin receptors [24, 52, 56, 68, 69]. Based on these observations, it is suggested that there may be several mechanisms by which the coronary artery causes coronary spasms [22, 23, 29, 56, 70–73]. A previous study reported that ACh provoked more diffuse and distal coronary spasms, whereas ER provoked more focal and proximal ones [74], indicating that ACh-provocation test might not provoke coronary spasm in all patients with coronary spasm. Further studies of the mechanisms underlying the difference between provoked-spasm sites in the ACh and ER provocation tests are needed.

## Diagnosis of coronary spasm by the ACh-provocation test

Studies in Japan have made many contributions to the pathophysiology, diagnosis, and treatment of coronary spasm, leading to the development of the Guidelines for Diagnosis and Treatment of Patients with Vasospastic Angina by the JCS [25, 54, 59, 75–78]. Although previous reports indicated that the prevalence of coronary spasm has been less in Western countries than in Asian (including Japan), recent reports have showed that the prevalence of coronary spasm in Western countries may be higher than indicated in previous reports [6, 10, 15, 79, 80].

**Fig. 2:**
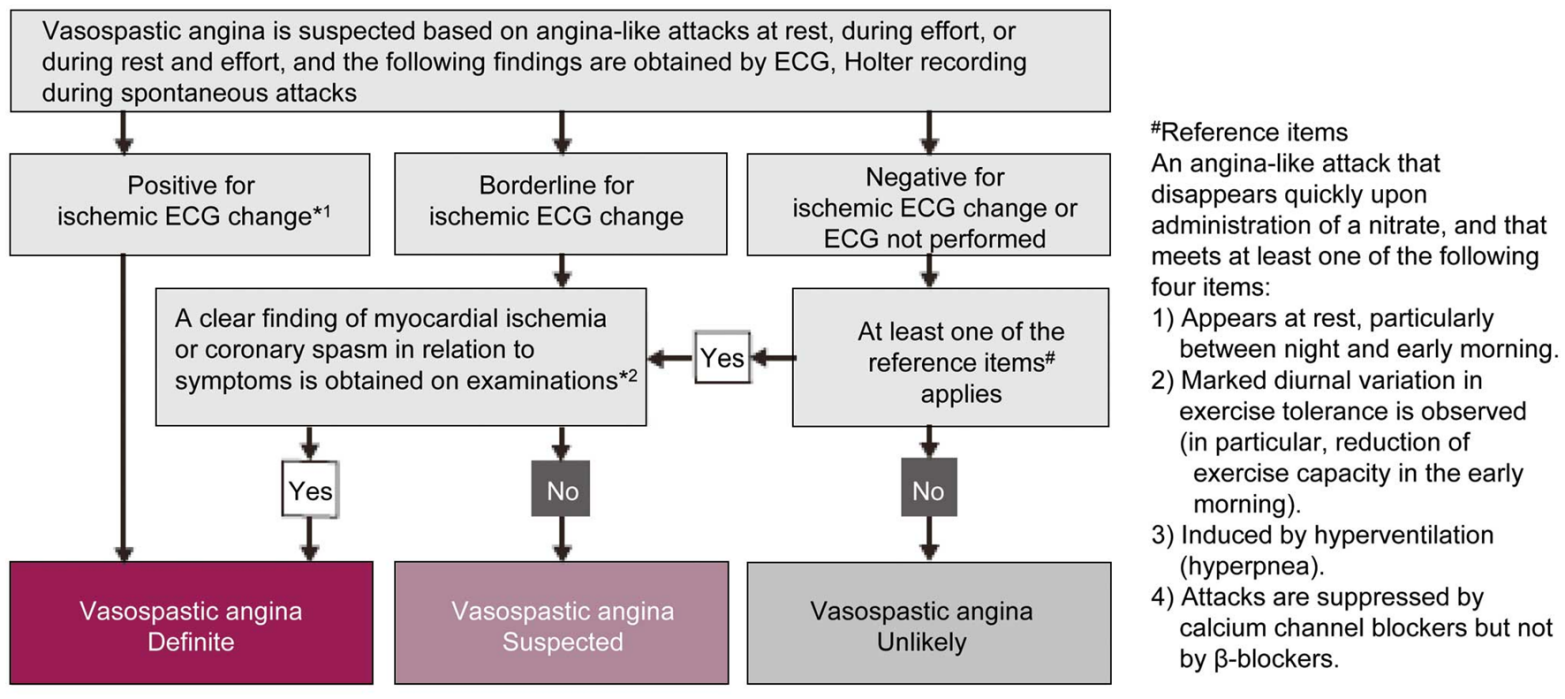
Diagnostic algorithm of vasospastic angina (coronary spastic angina). (Reproduced with permission from JCS Joint Working Group. Circ J 2014; 78: 2779–801 [25]). *1: Ischemic change is defined as a transient ST elevation of 0.1 mV or more, an ST depression of 0.1 mV or more, or new appearance of negative U waves, recorded in at least two contiguous leads on the 12-lead ECG. If the ischemic ECG change is prolonged, patients should be treated as directed in the guidelines for management of acute coronary syndrome. *2: Examinations include the drug-induced coronary spasm provocation test during cardiac catheterization and hyperventilation test. A positive finding for coronary spasm on coronary angiography in the acetylcholine- or ergonovine-induced coronary spasm provocation test is defined as “transient, total, or sub-total occlusion (> 90% stenosis) of a coronary artery with signs/symptoms of myocardial ischemia (anginal pain and ischemic ECG change)”

According to the JCS Guidelines for Vasospastic Angina (Coronary Spastic Angina) [25] (Fig. 2), we can consider a definitive diagnosis of coronary spasm when one of the following is true: (1) ischemic change is clearly observed on the ECG during attacks; (2) the ECG findings during an attack of coronary spasm are borderline but a clear finding of myocardial ischemia or coronary spasm is obtained in examinations (ACh- or ER-induced coronary spasm provocation test during CAG or hyperventilation test) and the patient has a history and symptoms during attacks that are consistent with coronary spasm; or (3) if there is no ECG change during a spontaneous attack of coronary spasm or if ECG examination has not been performed, at least one of the reference items (Fig. 2) is met, and this examination reveals a clear finding of myocardial ischemia or coronary spasm. A positive finding for coronary spasm on CAG in the ACh- or ER-induced coronary spasm provocation test is defined as “transient, total, or sub-total occlusion (> 90% stenosis) of a coronary artery with signs/symptoms of myocardial ischemia (anginal chest pain and ischemic ECG change)”. Because we do not often observe a spontaneous attack of coronary spasm in which ischemic change is clearly documented on the ECG, adding the invasive diagnostic ACh-provocation test can be useful to diagnose coronary spasm during CAG in patients with suspected coronary spasm [5, 16, 25].

**Fig. 3:**
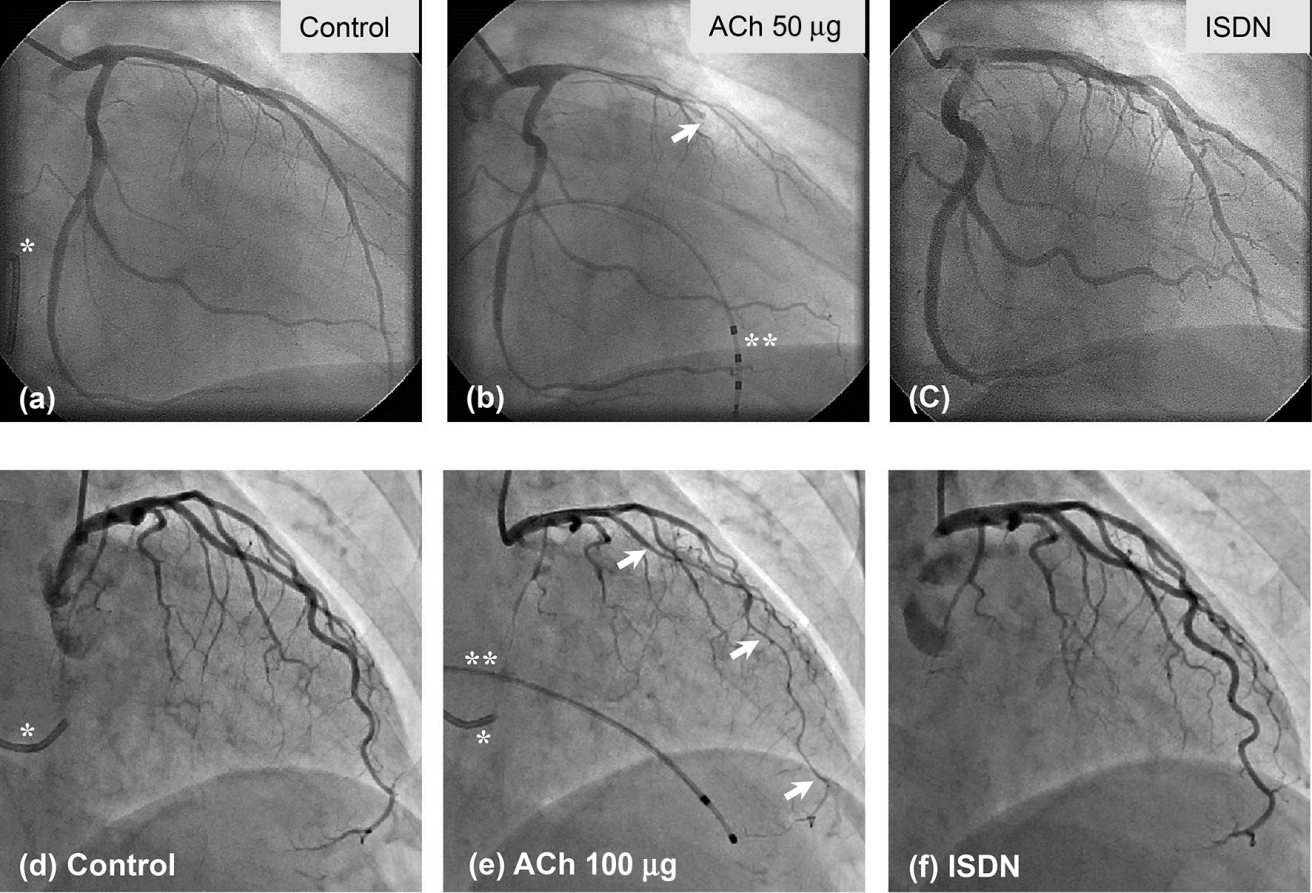
Acetylcholine (ACh)-provoked focal and diffuse spasm. Figures (a-c) show the focal spasm pattern. **a–c** Left coronary angiogram performed as a control before the ACh-provocation test. **b** Injection of ACh 50 μg into the LCA provoked complete occlusion in the LAD at the arrow position. **c** Injection of ISDN into the LCA improved the focal spasm in the LAD. Figures (**d–f**) show the diffuse spasm pattern. **d** Left coronary angiogram performed as a control before the ACh-provocation test. **e** Injection of ACh 100 μg into the LCA provoked diffuse spasm in the LAD. **f** Injection of ISDN into the LCA improved the diffuse spasm in the LAD. *ACh* acetylcholine, *LCA* left coronary artery, *LAD* left anterior descending coronary artery, *ISDN* isosorbide dinitrate. *Coronary sinus catheter, **Pacing catheter

We and others previously reported different patterns of angiographic changes including focal (total or sub-total obstruction) and diffuse spasm (severe diffuse vasoconstriction) during the spasm provocation test [52, 59, 60, 79, 81, 82]. In a retrospective, observational, and single-center 20-year study of consecutive patients who underwent the ACh-provocation test in our institution [54] (Fig. 3), ACh-provoked coronary spasm was observed in 873 (50%) of 1760 patients with typical or atypical angina-like chest pain (with 487/873 [56%] male, 386/873 [44%] female). Among the ACh-provoked coronary spasm patients, the number of focal spasms was 511/873 (59%) (with 343/511 [67%] male, 168/511 [33%] female), while the number of diffuse spasms was 362/873 (41%) (with 144/362 [40%] male, 218/362 female [60%]). We showed that female sex and low comorbidity of epicardial coronary artery stenosis (≥ 75%) were correlated with the ACh-provoked diffuse spasm pattern in patients with coronary spasm, and that ACh-provoked diffuse coronary spasm had a better prognosis than focal spasm for major adverse cardiac events (MACE). Another previous report showed that mixed (focal + diffuse) type multivessel coronary spasm had an important association with MACE [59]. These results suggest that we need to perform an ACh-provocation test to identify the subtype of ACh-provoked coronary spasm and to assess the risk of future cardiovascular events. Although the ACh-provoked diffuse spasm pattern is not at present included as a positive criterion for the ACh-provocation test in the JCS Guidelines [25], we may need to establish additional diagnostic criteria for coronary spasm in the future.

According to the JCS Guidelines, ACh-provocation test is not recommended during emergent CAG in patients with ACS [25]. The American Heart Association and American College of Cardiology guidelines also indicated that intracoronary provocative tests may be undertaken after ACS has become stable [83, 84]. However, some recent reports showed that during emergent CAG, ACh-provocation test was safely performed in ACS patients with nonobstructive culprit lesions and was useful to diagnose coronary spasm-induced ACS in those patients [6, 7, 85]. Nonetheless, the indication for the ACh-provocation test during emergent CAG in ACS patients remains controversial.

Epicardial coronary spasm can be seen angiographically during the ACh-provocation test; however, it is unclear whether the coronary spasm causes myocardial ischemia. For this reason, we usually measure myocardial lactate production in the coronary circulation during ACh-provocation tests in the left coronary artery as a supporting diagnostic marker for the evaluation of coronary spasm-induced myocardial ischemia [25, 86]. Our previous reports showed that ACh-provoked myocardial lactate production was more frequently observed in patients with diffuse spasm than in those with focal spasm [54], and that female sex, diabetes, and the − 786*T*/*C* single nucleotide polymorphism in endothelial nitric oxide synthase gene were associated with ACh-provoked myocardial lactate production in patients with coronary spasm [86]. Evaluation of the myocardial lactate production during ACh-provocation test might improve our diagnostic approach to nonobstructive CAD and our understanding of its pathophysiology.

## ACh-provoked coronary spasm and organic coronary artery stenosis

Coronary spasm can occur at sites of varying arteriosclerosis severity. We and others previously reported that coronary spasm could be associated with the progression of atherosclerosis [87–91]. Patients with coronary spasm are generally considered to have a good prognosis for cardiovascular events [54, 92–95]. On the other hand, the presence of organic coronary artery stenosis could be a significant prognostic factor for cardiovascular events in patients with coronary spasm [15, 78, 96–98]. Previous study reported that intermediate fixed coronary artery stenosis at the site of spasm provoked by ER could be a prognostic factor for cardiovascular events in patients with coronary spasm [99]; however, this study did not compare patients with coronary spasm occurring at sites other than the site of significant stenosis to patients with coronary spasm but without stenosis.

We previously reported that ACh-provoked coronary spasm occurring at the site of significant coronary artery stenosis was a more significant prognostic factor for MACE than ACh-provoked coronary spasm occurring at the site other than the site of organic stenosis, suggesting that coronary spasm can be associated with the cause of MACE when the coronary spasm occurs at the site of organic stenosis [100, 101]. This finding emphasized the need to identify the site of coronary spasm in patients with significant organic coronary artery stenosis to provide optimal medical treatment for coronary spasm patients with atherosclerotic disease.

**Fig. 4:**
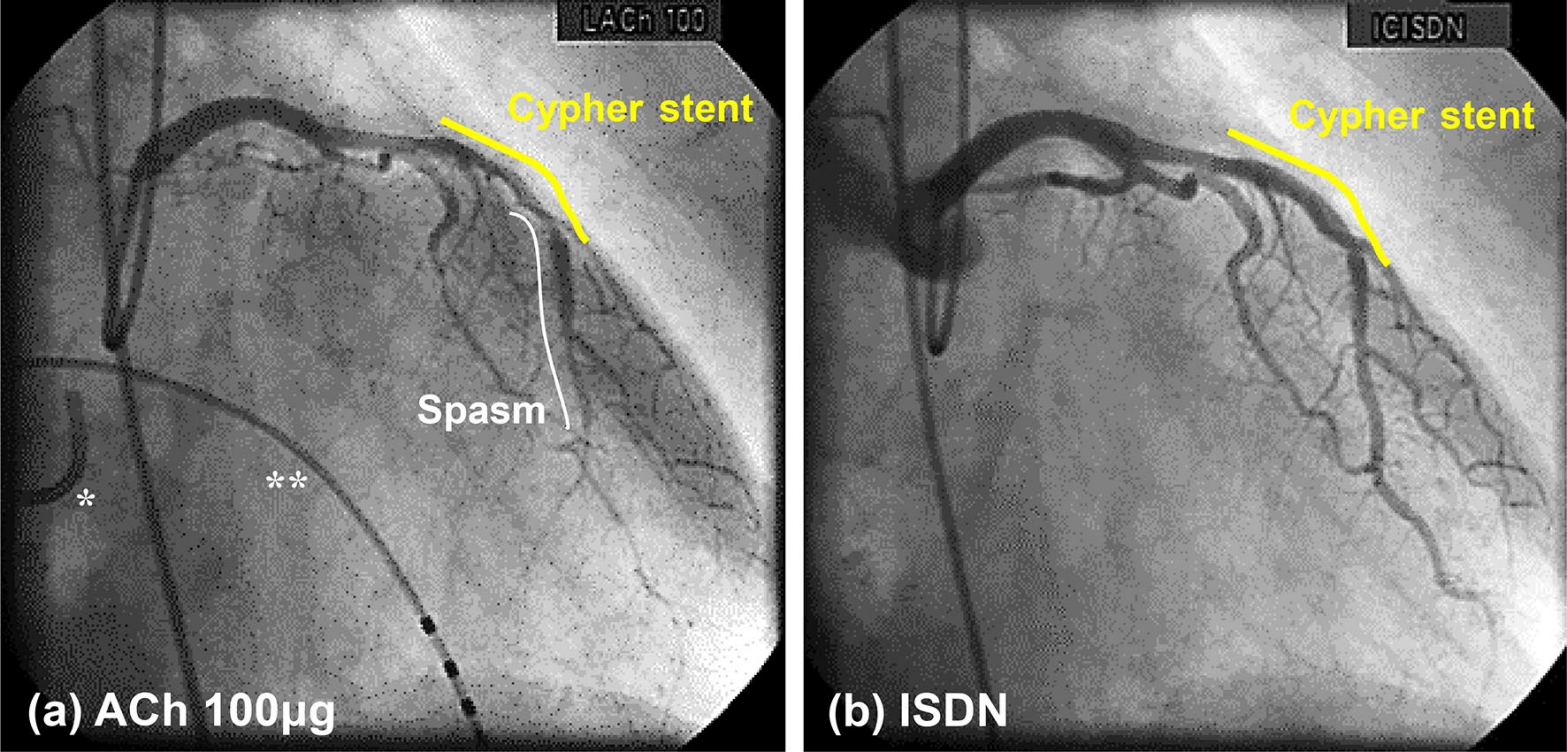
Coronary spasm after coronary stenting. **a** Injection of ACh 100 μg into the LCA provoked coronary spasm in LAD#7 stenting site with Cypher^®^. **b** Injection of ISDN into the LCA released coronary spasm and showed no in-stent restenosis and significant organic stenosis. *Ach* acetylcholine, *LCA* left coronary artery, *LAD* left anterior descending coronary artery, *ISDN* isosorbide dinitrate. *Coronary sinus catheter, **Pacing catheter

At present, we often perform PCI with coronary stent implantation for significant organic coronary artery stenosis. Coronary spasm occurring after coronary stent implantation has become a clinical problem [25, 102–107]. A previous report showed that excessive coronary vasoconstriction of PCI-treated coronary arteries in response to intracoronary injection of ACh was more frequent in patients treated with bare metal stents (BMS) than in those treated only with balloon angioplasty, suggesting that endothelial dysfunction induced by PCI was involved in the progression of atherosclerosis [108]. Furthermore, some reports showed that following intracoronary injection of ACh, vasoconstriction in segments adjacent to stents was more severe for drug-eluting stents (DES) than for BMS-treated coronary arteries [109, 110]. Regarding this point, we reported that second-generation DES occurred fewer cardiovascular events related to coronary spasm than first-generation DES or BMS because of the advanced design features of second-generation DES (higher biocompatibility and/or bioresorbable polymers) [103, 104]. These studies suggested that ACh-provocation test could be useful to assess the coronary vasoconstrictor response after coronary stent implantation (Fig. 4).

## Significance of the ACh spasm provocation test in left ventricular dysfunction

Recent studies reported that epicardial coronary spasm could be associated with left ventricular (LV) dysfunction [38–41]. We previously reported a case in which asymptomatic coronary spasm may have caused irreversible cardiac damage leading heart failure, suggesting that the presence or absence of coronary spasm in heart failure patients should be clarified to determine therapeutic strategy [40]. Several studies reported that multivessel or diffuse coronary spasm could lead to LV dysfunction such as dilated cardiomyopathy (DCM), and that the LV dysfunction in these patients was significantly improved following the administration of calcium channel blockers (CCBs) [38, 39, 41].

A previous study reported that the ACh-provocation test induced coronary spasm in 20/42 patients (48%) who had idiopathic DCM-like LV dysfunction without significant organic coronary arteries stenosis [38]. Patients in the ACh-positive (coronary spasm-induced) group were more likely to show ischemic changes on the ECG and were less likely to have myocardial fibrosis or degeneration on cardiac magnetic resonance imaging (MRI) or endomyocardial biopsy than patients in the ACh-negative (no coronary spasm) group. In the ACh-positive group, LV dysfunction was significantly improved following the administration of CCBs. This study suggested that repeated coronary spasm or persistent increase of coronary vasotonus could be one of the mechanisms leading to LV dysfunction, and that CCBs could improve LV dysfunction. In the ACh-positive group, MRI and endomyocardial biopsy showed no fibrous changes or degeneration. These results suggested that myocardial hibernation or stunning by repeated coronary spasm or persistent increase of coronary vasotonus leading to LV dysfunction might occur, and that CCBs might improve LV dysfunction by reducing the coronary spasm or coronary vasotonus.

On the other hand, ACh-provoked coronary vasomotor abnormality (e.g. epicardial coronary spasm or microvascular spasm) in our previous study was shown to be closely associated with myocardial fibrosis (expressed as late gadolinium enhancement in cardiac MRI) in patients with heart failure and without organic coronary artery stenosis [111, 112]. Coronary vasomotor abnormalities such as coronary spasm or microvascular spasm could affect the development of nonobstructive CAD, and might also be associated with LV dysfunction and heart failure [4, 19, 54, 113–117]. It is suggested that coronary vasomotor abnormality diagnosed by ACh-provocation test could be involved in myocardial fibrosis and worsening heart failure. Although it is still controversial whether coronary spasm or microvascular spasm might contribute to the myocardial fibrosis, ACh-provocation test could provide a detailed pathophysiology of LV dysfunction with nonobstructive CAD, and might be useful to determine therapeutic strategy in patients with unknown cause of LV dysfunction.

## Significance of microvascular spasm in nonobstructive coronary artery disease

The coronary arterial tree consists of not only the epicardial coronary arteries, but also smaller arteries and arterioles (< 500 μm). The latter feed the capillaries and play an important role in the coronary microcirculation, being the main site of regulation of myocardial blood flow [113, 118, 119]. Epicardial coronary arteries without significant stenosis can contribute only about 5% of total coronary resistance, suggesting that coronary microvessels can play an important role in regulating myocardial blood flow [25]. The importance of microvascular function has been recognized because patients with impaired microvascular function had worse outcomes following cardiovascular events than those with normal microvascular function [76, 120–123].

Modalities for diagnosing obstructive CAD such as CAG or CTA have improved remarkably. However, in patients with suspected angina, we can sometimes see both nonobstructive epicardial coronary arteries and non-ACh-provoked epicardial coronary spasm. Nonobstructive CAD has included not only angina with epicardial coronary spasm but also microvascular spasm or dysfunction [5–7, 62, 120, 121, 124] (Fig. 1). The prevalence of microvascular spasm or dysfunction in patients with nonobstructive CAD is unknown even after exclusion of epicardial coronary spasm [4, 10, 62, 118, 125]. One of the reasons may be because we have no standard diagnostic criteria for microvascular spasm or dysfunction.

**Fig. 5:**
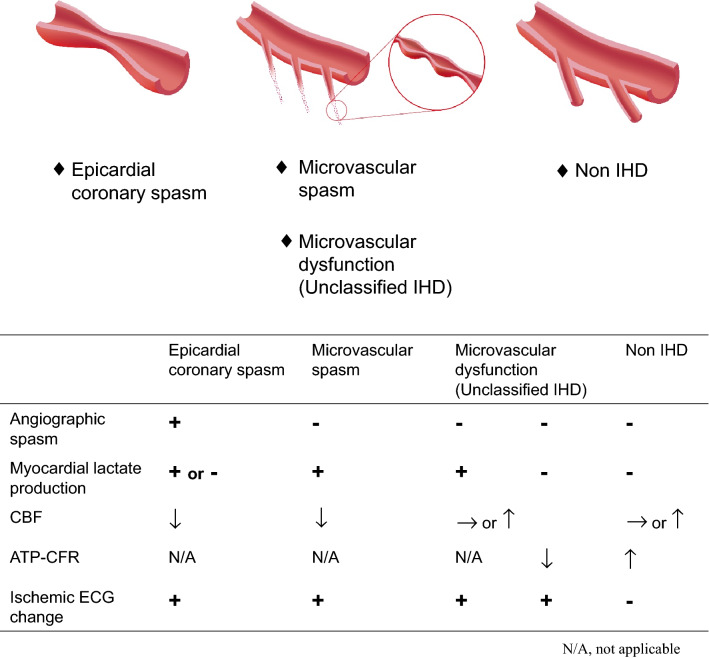
A categorization scheme for functional evaluation using ACh-provocation test in the nonobstructive CAD patients. This scheme shows the categorization of epicardial coronary artery spasm, microvascular spasm, microvascular dysfunction (unclassified IHD), and non-IHD by the intracoronary ACh-provocation test, simultaneous measurement of myocardial lactate production, quantitative CBF, and ATP-CFR in nonobstructive CAD patients. *Ach* acetylcholine, *CAD* coronary artery disease, *IHD* ischemic heart disease, *CBF* coronary blood flow, *ATP-CFR* adenosine triphosphate-induced coronary flow reserve, *ECG* electrocardiogram

We previously reported the possibility of diagnosing microvascular spasm or dysfunction by a combination of intracoronary ACh-provocation test, simultaneous measurement of myocardial lactate production, quantitative CBF, and ATP-CFR in patients with suspected angina with nonobstructive CAD [4] (Fig. 5). We defined microvascular spasm as meeting both of the following 2 criteria: (1) positive for myocardial lactate production and (2) decreased CBF during ACh-provocation test in patients with suspected angina and non-ACh-provoked epicardial coronary spasm. We defined microvascular dysfunction as meeting both of the following two criteria: (1) positive for myocardial lactate production and (2) non-decreased CBF in patients with suspected angina and non-ACh-provoked epicardial coronary spasm. In addition, we also defined microvascular dysfunction as meeting both of the following two criteria: (1) negative for myocardial lactate production and (2) decreased ATP-CFR. We categorized those patients with microvascular dysfunction to the unclassified ischemic heart disease (IHD) group. In this report, among 154 patients with suspected angina having non-ACh-provoked epicardial coronary spasm (with 47/154 [31%] male, 107/154 [69%] female), microvascular spasm was observed in 50/154 (32%) of patients (with 5/50 [10%] males, 45/50 [90%] females), and microvascular dysfunction (unclassified IHD) was observed in 32/154 (21%) of patients (with 9/32 [28%] males, 23/32 [72%] females). Some previous reports showed that during the ACh-provocation test, the coronary blood supply (as determined by quantitative CBF with an intracoronary doppler-tipped guidewire [FloWire]) could be associated with microvascular function [4, 62, 118, 126–133]. The ACh-provocation test can be useful to assess the reactivity (such as spasm) of not only of epicardial coronary arteries but also endothelium-dependent coronary microvessels. In addition, ATP-CFR is a tool to test the non-endothelium-dependent coronary artery reactivity and can be useful to assess the microvascular function in nonobstructive CAD [4, 63, 127, 128, 134]. Using several modalities during the ACh-provocation test, such as measurement of CBF, myocardial lactate production, and ATP-CFR, we can assess several types of nonobstructive CAD such as epicardial coronary spasm, microvascular spasm, and microvascular dysfunction in the suspected angina patients with nonobstructive CAD in routine clinical cardiovascular practice (Fig. 5).

Our report mentioned above [4] of the 154 patients with suspected angina and non-ACh-provoked epicardial coronary spasm showed that female sex, a lower body mass index, minor-borderline ischemic ECG changes at rest, limited baseline diastolic-to-systolic velocity ratio of CBF, and attenuated ATP-CFR were significantly associated with microvascular spasm. In this report, the prevalence of microvascular spasm (50/154, 32%) was higher in females than males (with 45/50 [90%] vs 5/50 [10%], respectively), and the average age and body mass index of patients with microvascular spasm were 62.7 ± 10.6 years and 22.3 ± 2.9 kg/m^2^, respectively. These clinical features of the microvascular spasm, such as female sex, middle age, and low body mass index, are reminiscent of postmenopausal phenomena, which invites speculation of an association between the pathogenesis of microvascular spasm and age-dependent estrogen deficiency in those patients.

## Limitations and complications in ACh-provocation test

The ACh-provocation test has several limitations. The ACh-provocation test cannot provoke coronary spasm in all patients with coronary spasm, although our previous study showed high sensitivity and specificity of the test in patients with variant angina [20, 21]. In patients with chronic kidney disease, the use of contrast medium during ACh-provocation test can have adverse effects. Radiation exposure during the test can be increased because of multiple procedures of the ACh-provocation test [56]. Finally, the ACh-provocation test is not recommended if the patient has severe bronchial asthma [56, 135].

ACh-provocation test has several potential complications. In our institution, the number of life-threatening arrhythmias during ACh-provocation test was 9 (0.5%) of 1637 patients who underwent this test [54]. A previous report showed that the number of serious major complications, such as sustained ventricular tachycardia, cardiac tamponade, and shock, was 4 (0.6%) of 715 patients during ACh-provocation test, but with no deaths occurring [55]. Other reports also showed that the incidence of complications during ACh-provocation test can be low [6, 8, 24, 53, 58, 59, 74, 136]. Coronary spasm induced by ACh-provocation test can be susceptible to spontaneous amelioration because the pharmacological effect of ACh is short [9, 22]. Based on these observations, ACh-provocation test can be a safe method, although it should be performed carefully.

## Conclusions and future perspectives

As summarized in this review, ACh-provocation test can be a safe and reliable method not only to diagnose epicardial coronary spasm but also to assess the pathophysiology of nonobstructive CAD. The ACh-provocation test is not often performed in routine clinical cardiovascular practice because multiple procedures of this test are required. However, the pathophysiology showed by ACh-provocation test can be associated with prognosis in patient with nonobstructive CAD. We can identify the high-risk patient with coronary spasm or microvascular spasm diagnosed by ACh-provocation test and treat with intensive care for the patients.

We hope that cardiologists will routinely perform the ACh-provocation test to assess the pathophysiology of nonobstructive CAD.
